# Trends in prostatectomy utilization: Increasing upfront prostatectomy and postprostatectomy radiotherapy for high‐risk prostate cancer

**DOI:** 10.1002/cam4.3482

**Published:** 2020-10-31

**Authors:** Bryan Kaps, Michael Leapman, Yi An

**Affiliations:** ^1^ Department of Urology Yale University School of Medicine New Haven CT USA; ^2^ Yale Cancer Center New Haven CT USA; ^3^ Department of Therapeutic Radiology Yale University School of Medicine New Haven CT USA

**Keywords:** Adjuvant Radiation Therapy, Prostate Cancer, Prostatectomy, Radiation, Treatment trends

## Abstract

We aimed to determine patterns in frequency of radiotherapy for prostate cancer and definitive surgical management. There is prospective evidence indicating benefits of radiotherapy for some patients after radical prostatectomy (prostatectomy), with recent evidence suggesting benefit of early salvage radiotherapy. Trends in postoperative radiotherapy have not been elucidated. We analyzed the National Cancer Database for prostate cancer patients treated with curative‐intent therapy between 2004 and 2016. Patients were risk stratified according to NCCN treatment guidelines. Linear regression was utilized to examine trends in treatment with initial prostatectomy and trends in postoperative radiotherapy among treatment risk groups. Multivariable logistic regression was utilized to examine clinical‐demographic variables associated with prostatectomy and postoperative radiotherapy. From 2004 to 2016, 508,450 patients received prostatectomy and 370,314 received radiotherapy. Median age was 63.6 years. There was increased utilization of prostatectomy from 47.9% in 2004 to 61.3% in 2016 (p_trend_ <0.001). 24,466 cases received postoperative radiotherapy. Similarly, postoperative radiotherapy utilization increased from 2.2% in 2004 to 4.0% in 2016 (p_trend_ <0.001). The subgroup with the largest increase in postoperative radiotherapy was clinically high‐risk disease (5.3% in 2004 to 7.8% in 2016 (p_trend_ <0.001). Clinical high‐risk disease (OR 1.751), Gleason 9‐10 (OR 2.973), and PSA >20 ng/ml (OR 1.489) were factors predictive for postoperative radiotherapy. The proportion of prostate cancer patients who undergo definitive prostatectomy and postoperative radiotherapy is increasing. This increase is greatest in high‐risk cases. Overall, the proportion of patients who receive any radiotherapy is decreasing. Association with preclinical factors suggests optimization of patient selection should be considered.

## Graphical abstract

Utilization of prostatectomy for primary treatment of local prostate cancer has been increasing. With this increased utilization of prostatectomy, an increase has also been seen in the number of patients receiving postoperative radiation therapy, principally among patients with clinically high‐risk local prostate cancers.

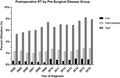

## 1 BACKGROUND

Prostate cancer (PCa) is the most common non‐cutaneous cancer in men, with either definitive radiotherapy (RT) and radical prostatectomy (RP) as standard of care curative option for those with non‐metastatic disease.^1^ Each therapy is associated with different complications and risks, and therapy choice is largely individualized to patients after discussion with providers.^2^, ^3^

Despite this, many studies are attempting to compare the benefits of RT or RP for primary treatment in patients with PCa. Most recently, the focus has been on comparing treatment benefits in patients with high‐risk clinical features of their PCa, which have shown mixed result.^4^, ^5^ Although results have been mixed, other studies have shown a significant increase in the number of patients receiving RP each year in comparison to RT.^6^ For many patients, RP constitutes a cure, though, in a small minority with local recurrence or high‐risk pathologic features, adjuvant or salvage RT is eventually recommended.^7^, ^8^, ^9^ Although suggested in these groups, studies suggest adjuvant and salvage RT is underutilized for these patients with adverse features.^10^ In other subgroups of interest, such as clinically high‐risk patients, trends in postoperative RT have not yet been elucidated.

Here, we utilized the National Cancer Database (NCDB) to identify patients receiving curative treatment for PCa. We evaluated changing utilization patterns of patients who received RT or RP as primary treatment. For patients who received surgery as primary treatment, we additionally assessed changing patterns in RT utilization after surgery. We investigated clinical and demographic factors associated with increased likelihood of receiving upfront RP as well as postoperation RT.

## 2 METHODS

**Figure 1:**
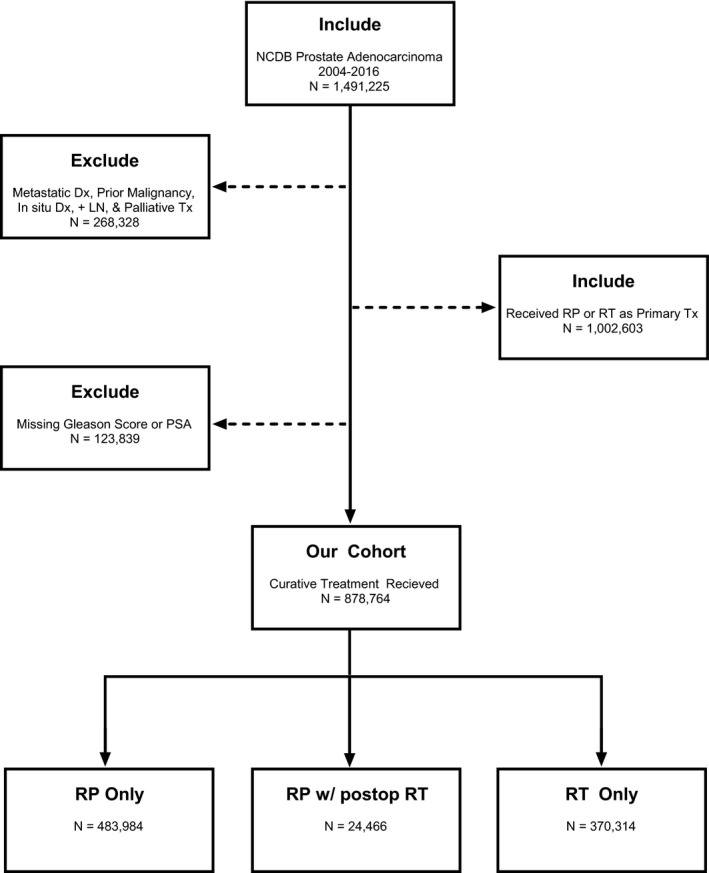
Study inclusion and exclusion criteria are illustrated. +LN, Positive regional lymph nodes; Dx, Disease; NCDB, National Cancer Database; PSA, Prostate‐Specific Antigen; RP, Radical Prostatectomy; RT, Radiation Therapy; Tx, Treatment

The National Cancer Database (NCDB) is the largest registry of patients with a primary diagnosis of cancer, capturing over 70% of all newly diagnosed cancers in the United States.^11^ PCa specific details such as prostate‐specific antigen and Gleason score and pattern are included. More extensive discussion of variables in the database has previously been described.^12^ The database is made available by the joint program of the Commission of cancer for the American College of Surgeons and the American Cancer Society, and is exempt from our institutional review board as a publicly available and deidentified database. The inclusion and exclusion criteria are summarized in Figure 1. We included patients >18 years of age with PCa treated with curative intent prostatectomy or radiotherapy and excluded metastatic disease and prior malignancy. Additionally, cases were excluded if they were missing Gleason, PSA, or incomplete risk categorization information before primary treatment.

Patients were divided into three treatment classifications: radiation only, surgery without subsequent curative radiation, and surgery with postoperative radiation. Patients who received curative radiation after surgery were included under the last treatment classification. Patients were further classified as clinically low, intermediate, and high‐risk PCa based on the 2016 NCCN guidelines.^3^ High‐risk patients had either a Gleason 8‐10, PSA >20 ng/ml, or clinical T stage T3‐T4. Binary variables were categorized as follows: race (white, non‐white), year of diagnosis (2004–2010, 2011–2016), insurance (Noninsured, insured), treatment facility (nonacademic, academic), travel distance (<20 miles, ≥20 miles), comorbidities (Charlson‐Deyo Score 0, ≥0), and facility volume (high, low). A high‐volume facility volume was defined as an institution that was in the 90th percentile when considering total number of PCa cases each institution had in the database, as previously done.^13^

Linear regression was used to determine the statistical significance of temporal trends in therapy utilization. Univariable logistic regression was used to determine binary variables that were associated with the receipt of surgery versus radiation, as well as the receipt of follow up radiation after surgery. Binary variables significant on univariable regression, all clinically relevant variables, and non‐binary variables of interest were included in a multivariable model for receipt of surgery and a multivariable model for follow‐up radiation after surgery. Statistical analysis was performed in STATA IC 13.1 (StataCorp, Texas, USA). A two‐sided *p* < 0.05 was considered statistically significant.

## 3 RESULTS

We identified 878,764 men with clinically localized PCa who received curative therapy for PCa between the years of 2004 and 2016. The mean age was 63.7 years, and 81.5% of patients was classified as White race. Of those receiving curative treatment for PCa, 21.8% was clinically high risk, 47.9% intermediate risk, and 30.3% was low risk (Table 1).

**Table 1:** Patient cohort characteristics

Variable	Total	Low Risk	Int Risk	High Risk
	*N* = 878,764 (100.0)	*N* = 266,092 (30.3)	*N* = 421,124 (47.9)	*N* = 191,548 (21.8)
Age, mean	63.7	62.0	63.7	66.0
	*N* (%)	*N* (%)	*N* (%)	*N* (%)
Treatment Modality				
Total Curative Tx	878,764 (100.0)	266,092 (100.0)	421,124 (100.0)	191,548 (100.0)
Rad Only	370,314 (42.1)	113,790 (42.8)	158,404 (37.6)	98,120 (51.2)
Surgery	508,450 (57.9)	152,302 (57.2)	262,720 (62.4)	93,428 (48.8)
w/o RT	483,984 (55.1)	150,592 (56.6)	252,909 (60.1)	80,483 (42.0)
w/ RT	24,466 (2.8)	1710 (0.6)	9811 (2.3)	12,945 (6.76)
Race				
White	716,057 (81.5)	222,011 (83.4)	341,981 (81.2)	152,065 (79.4)
Non‐white	162,707 (18.5)	44,081 (16.6)	79,143 (18.8)	39,483 (20.6)
Diagnosis Year				
2004	65,009 (7.4)	24,911 (9.4)	27,613 (6.6)	12,485 (6.5)
2005	66,621 (7.6)	25,021 (9.4)	29,011 (6.9)	12,589 (6.6)
2006	74,456 (8.5)	27,423 (10.3)	33,311 (7.9)	13,722 (7.2)
2007	80,022 (9.1)	28,366 (10.7)	37,244 (8.8)	14,412 (7.5)
2008	77,199 (8.8)	24,843 (9.3)	37,899 (9.0)	14,457 (7.6)
2009	64,281 (7.3)	19,040 (7.2)	32,865 (7.8)	12,376 (6.5)
2010	72,856 (8.3)	25,180 (9.5)	32,896 (7.8)	14,780 (7.7)
2011	75,234 (8.6)	24,863 (9.3)	34,823 (8.3)	15,548 (8.1)
2012	61,429 (7.0)	17,349 (6.5)	29,881 (7.1)	14,199 (7.4)
2013	59,735 (6.8)	14,781 (5.6)	30,080 (7.1)	14,874 (7.8)
2014	57,157 (6.5)	12,546 (4.7)	29,136 (6.9)	15,475 (8.1)
2015	62,279 (7.1)	11,741 (4.4)	32,854 (7.8)	17,684 (9.2)
2016	62,486 (7.1)	10,028 (3.8)	33,511 (8.0)	18,947 (9.9)
Clinical T Stage				
T1	549,528 (62.5)	205,086 (77.1)	251,169 (59.6)	93,273 (48.7)
T2	21,1091 (24.0)	25,910 (9.7)	127,385 (30.3)	57,796 (30.2)
T3/T4	23,931 (2.7)	0 (0.0)	0 (0.0)	23,931 (12.5)
Missing	94,214 (10.7)	35,096 (13.2)	42,570 (10.1)	16,548 (8.6)
Gleason Score				
2‐6	34,4768 (39.2)	266,092 (100.0)	59,367 (14.1)	19,309 (10.1)
7	40,0257 (45.6)	0 (0.0)	361,757 (85.9)	38,500 (20.1)
8‐9	78,203 (8.9)	0 (0.0)	0 (0.0)	78,203 (40.8)
10	55,536 (6.3)	0 (0.0)	0 (0.0)	55,536 (29.0)
PSA Level				
<10 ng/ml	69,3622 (78.9)	266,092 (100.0)	338,184 (80.3)	89,346 (46.6)
10‐20 ng/ml	113,740 (12.9)	0 (0.0)	82,940 (19.7)	30,800 (16.1)
>20 ng/ml	71,402 (8.1)	0 (0.0)	0 (0.0)	71,402 (37.3)
Income				
<35 K	133,034 (15.2)	37,906 (14.3)	62,982 (15.0)	32,146 (16.9)
≥ 35 K	741,557 (84.8)	226,898 (85.7)	356,195 (85.0)	158,464 (83.1)
Insurance Status				
Private	458,964 (53.7)	158,982 (61.3)	219,356 (53.5)	80,626 (43.5)
Government	395,417 (46.3)	100,225 (38.7)	190,551 (46.5)	104,641 (56.5)
Treatment Facility Type				
Non‐academic	545,175 (62.1)	167,655 (63.1)	256,963 (61.1)	120,557 (63.0)
Academic	333,012 (37.9)	98,140 (36.9)	163,932 (39.0)	70,940 (37.0)
Travel Distance				
<20 miles	582,442 (66.5)	174,852 (66.0)	275,920 (65.7)	131,670 (69.0)
≥20 miles	293,185 (33.5)	90,253 (34.0)	143,765 (34.3)	59,167 (31.0)
Charlson‐Deyo Score				
0	739,087 (84.1)	228,996 (86.1)	351,734 (83.5)	158,357 (82.7)
≥1	139,677 (15.9)	37,096 (13.9)	69,390 (16.5)	33,191 (17.3)
Facility Volume				
Low volume	545,175 (62.1)	189,367 (71.2)	299,072 (71.0)	145,790 (76.1)
High volume	333,012 (37.9)	76,725 (28.8)	122,052 (29.0)	45,758 (23.9)
US Census Region				
Northeast	190,107 (21.7)	58,753 (22.1)	91,158 (21.7)	40,196 (21)
Midwest	308,871 (35.2)	97,246 (36.6)	143,930 (34.2)	67,695 (35.4)
South	234,450 (26.7)	69,794 (26.3)	113,311 (26.9)	51,345 (26.8)
West	144,759 (16.5)	40,002 (15.1)	72,496 (17.2)	32,261 (16.9)

### 3.1 Trends in Upfront Radical Prostatectomy

**Figure 2:**
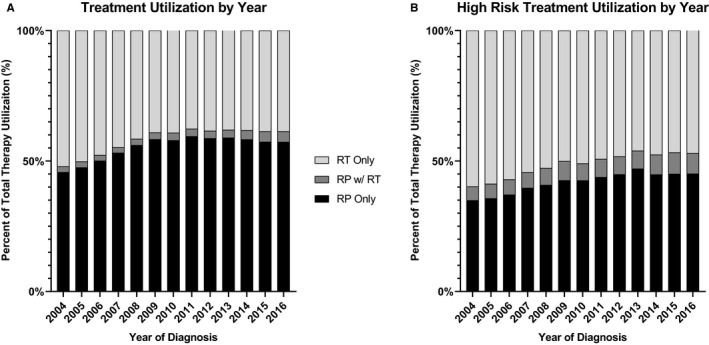
Treatment patterns for curative therapy, with postoperative radiation therapy superimposed on pertinent radical prostatectomy cases. (A) Entire cohort and (B) High‐risk disease. RP, radical prostatectomy; RT, radiation therapy

Of the total cases, 508,450 (57.9%), were treated with RP as primary treatment, and 370,314 (42.1%) were treated with RT as primary treatment. The number of cases utilizing RP for primary treatment of PCa increased from 2004 to 2016. RP utilization was used in 47.9% of cases as primary treatment in 2004, which increased to 61.3% of cases in 2016 (p_trend_ <0.001), see Figure 2. As such, utilization of RT for treatment steadily decreased by an equal percentage, with 52.1% of cases receiving RT in 2004 to 38.2% of cases in 2016.

When stratified by disease risk category (low, intermediate, and high risk), the trends concerning RP and RT utilization were similar. RP utilization increased in all disease categories and corresponded to a decrease in radiation utilization. For the high‐risk disease category, 40.2% of cases utilized RP as primary treatment in 2004, compared with 52.9% in 2016 (p_trend_ <0.001). For intermediate risk, the change was from 53.1% in 2004 to 64.3% in 2016 (p_trend_ =0.013), and for low risk, the change was from 45.9% in 2004 to 66.9% in 2016 (p_trend_ <0.001).

### 3.2 Use of RT after RP

**Figure 3:**
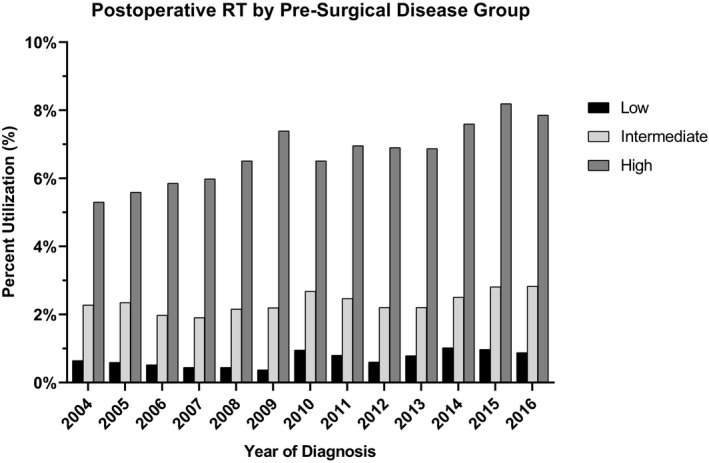
Postoperative treatment patterns between 2004 and 2016. Low, Intermediate, and High legend labels correspond to NCCN disease severities low risk, intermediate risk, and high risk. RT, Radiation Therapy

After RP, 2.2% of cases had utilization of postoperative RT in 2004 which increased to 4.0% of all cases in 2016 (p_trend_ <0.001) Figure 2A. When stratified by disease risk category the clinically high‐risk disease group had the most significant increases in radiation after surgery (Figure 3). For the high‐risk cases, the increase was from 5.3% of cases receiving RT after RP in 2004, to 7.8% of cases receiving RT after RP in 2016 (p_trend_ < 0.001) (Figure 2B). The intermediate and low‐risk disease group also had a statistically significant increase in postoperative radiation, but the magnitude of the increase was smaller than the high‐risk disease group. For the intermediate‐risk group, the utilization of postoperative RT increased from 2.3% of cases in 2004 to 2.8% in 2016 (p_trend_ = 0.015). For the low‐risk group, the utilization was 0.6% in 2004 to 0.9% in 2016 (p_trend_ = 0.010).

### 3.3 Factors associated with receipt of RP and management choice after RP

Univariable and multivariable regressions were performed to investigate factors that were predictive of receipt of either RP or RT as primary treatment, and eventual receipt of postoperative RT for the cases who received RP as primary treatment (Table 2). Advanced age (59+) was the strongest predictor for receiving RT rather than RP of the demographic variables (OR 0.381, *p* < 0.001). Non‐white race (OR 0.705, *p* < 0.001) and government insurance (OR 0.419, *p* < 0.001) were also associated with receipt of RT instead of RP. Demographic variables were positive predictors for the receipt of RP, such as higher income (OR 1.273, *p* < 0.001), academic facility (OR 1.307, *p* < 0.001), greater travel distance (OR 1.690, *p* < 0.001), comorbidities (OR 1.642, *p* < 0.001), and high‐volume facilities (OR 1.472, *p* < 0.001). Of the clinical variables, high PSA levels >20 ng/ml had strong negative predictors for receipt of RP (OR 0.360, *p* < 0.001) as well as high Gleason biopsy scores >8 (OR 0.554, *p* < 0.001) and clinical T stage 3/4 (OR 0.545, *p* < 0.001). In contrast, clinical high risk was a predictor of upfront surgery (OR 2.414, *p* < 0.001).

**Table 2:** Association between demographic, socioeconomic, and clinical factors with the receipt of radical prostatectomy for definitive treatment

Variable	Univariable Analysis		Multivariable Analysis	
	AOR (95% CI)	*p* value	AOR (95% CI)	*p* value
Age				
18–59	1.000	<0.001	1.000	<0.001
59+	0.270 (0.267–0.272)		0.381 (0.376–0.385)	
Race				
White	1.000	<0.001	1.000	<0.001
Non‐white	0.759 (0.751–0.767)		0.705 (0.695–0.714)	
Diagnosis Year				
2004–2010	1.000	<0.001	1.000	<0.001
2011–2016	1.310 (1.299–1.322)		1.725 (1.707–1.742)	
Clinical Risk Category				
Low			1.000	<0.001
Intermediate			1.646 (1.611–1.681)	
High			2.414 (2.322–2.508)	
Clinical T Stage				
T1			1.000	<0.001
T2			0.879 (0.868–0.889)	
T3/T4			0.545 (0.527–0.564)	
Gleason Score Core Biopsy				
2–6			1.000	
7			1.012 (0.993–1.032)	0.219
8			0.563 (0.542–0.584)	<0.001
9–10			0.554 (0.534–0.575)	<0.001
PSA Level				
<10 ng/ml			1.000	<0.001
10–20 ng/ml			0.576 (0.567–0.585)	
>20 ng/ml			0.360 (0.349–0.371)	
Income				
<35 K	1.000	<0.001	1.000	<0.001
≥35 K	1.403 (1.386–1.419)		1.273 (1.254–1.291)	
Insurance Status				
Private	1.000	<0.001	1.000	<0.001
Government	0.295 (0.292–0.297)		0.419 (0.414–0.423)	
Treatment Facility Type				
Non‐academic	1.000	<0.001	1.000	<0.001
Academic	1.694 (1.679–1.708)		1.307 (1.291–1.323)	
Travel Distance				
<20 miles	1.000	<0.001	1.000	<0.001
≥20 miles	1.939 (1.920–1.957)		1.690 (1.671–1.710)	
Charlson‐Deyo Score				
0	1.000	<0.001	1.000	<0.001
≥1	1.236 (1.222–1.251)		1.642 (1.62–1.663)	
Treatment Facility Volume				
Low volume	1.000	<0.001	1.000	<0.001
High volume	2.117 (2.096–2.138)		1.472 (1.452–1.492)	
US Census Region				
Northeast			1.000	<0.001
Midwest			1.202 (1.185–1.219)	
South			1.316 (1.297–1.335)	
West			1.448 (1.425–1.473)	

When evaluating factors associated with an increased probability of receiving postoperative RT, pT4 (OR 15.868, *p* < 0.001) was the strongest predictor (Table 3). Preoperative risk factors also predicted receipt of RT after RP, including clinical high risk (OR 1.754, *p* < 0.001) and clinical intermediate risk (OR 1.249, *p* < 0.001) disease groups, Gleason score 9‐10 (OR 2.954, *p* < 0.001), PSA >20 ng/ml (OR 1.488, *p* < 0.001), and cT3/4 (OR 1.165, *p* < 0.001). Demographic and socioeconomic variables age (OR 0.801, *p* < 0.001) and insurance (OR 0.947, *p* < 0.001) had much weaker predictors for utilization of RT after RP, than for RP as primary therapy. Of the socioeconomic factors, high volume facilities (OR 0.613, *p* < 0.001) and travel distance >20 miles (OR 0.628, *p* < 0.001) had strong negative predictors for radiation.

**Table 3:** Association between demographic, socioeconomic, and clinical factors with the receipt of radiation therapy after radical prostatectomy

Variable	Univariable Analysis		Multivariable Analysis	
	AOR (95% CI)	*p* value	AOR (95% CI)	*p* value
Age				
18–59	1.000	<0.001	1.000	<0.001
59+	0.665 (0.648–0.683)		0.801 (0.771–0.833)	
Race				
White	1.000		1.000	
Non‐white	0.961 (0.930–0.994)	0.019	1.029 (0.983–1.077)	0.217
Diagnosis Year				
2004–2010	1.000		1.000	<0.001
2011–2016	1.427 (0.391–1.464)		0.890 (0.859–0.923)	
Pathologic T Stage				
T2			1.000	<0.001
T3			9.414 (9.024–9.821)	
T4			15.868 (13.808–18.235)	
Clinical Risk Category				
Low			1.000	<0.001
Intermediate			1.249 (1.130–1.381)	
High			1.754 (1.558–1.975)	
Clinical T Stage				
T1			1.000	<0.001
T2			1.083 (1.044–1.125)	
T3/T4			1.165 (1.085–1.250)	
Gleason Score				
2–6			1.000	<0.001
7			1.487 (1.372–1.611)	
8			2.080 (1.878–2.303)	
9–10			2.954 (2.672–3.267)	
PSA Levl				
<10 ng/ml			1.000	<0.001
10–20 ng/ml			1.428 (1.367–1.491)	
>20 ng/ml			1.488 (1.397–1.585)	
Income				
<35 K	1.000	<0.001	1.000	
≥ 35 K	1.102 (1.062–1.143)		0.968 (0.921–1.018)	0.205
Insurance Status				
Private insurance	1.000	<0.001	1.000	
Government insurance	0.685 (0.667–0.704)		0.947 (0.912–0.984)	0.005
Treatment Facility Type				
Non‐academic	1.000	<0.001	1.000	<0.001
Academic	0.945 (0.921–0.971)		0.894 (0.860–0.929)	
Travel Distance				
<20 miles	1.000	<0.001	1.000	<0.001
≥20 miles	0.784 (0.763–0.807)		0.628 (0.605–0.652)	
Charlson‐Deyo Score				
0	1.000	<0.001	1.000	<0.001
≥ 1	1.090 (1.054–1.127)		0.896 (0.858–0.935)	
Treatment Facility Volume				
Low volume	1.000	<0.001	1.000	<0.001
High volume	0.768 (0.745–0.791)		0.613 (0.587–0.639)	
US Census Region				
Northeast			1.000	
Midwest			0.993 (0.945–1.042)	0.765
South			1.150 (1.096–1.206)	<0.001
West			1.138 (1.079–1.201)	<0.001

## 4 DISCUSSION

We observed that surgical intervention for the primary treatment of PCa has been increasing. Our updated observation is consistent with older studies that have also suggested increasing RP utilization.^6^ The implications of this increase in RP on patient care are not immediately clear. To date, no study has shown either RT or RP to be superior for the primary treatment of PCa. The ProtecT randomized control trial (RCT) did not find any difference between RT or RP in primary oncologic outcomes, but this study was designed more to compare active surveillance to primary treatment in intermediate to low‐risk patients.^14^ Nevertheless, we hypothesize that the increased utilization of RP may be impacting postoperative treatment trends. As treatment with RP has increased over the study period, the percentage of patients being treated with postoperative RT has increased as well. When patients choose RP, the general aim is to cure without additional adjuvant or salvage treatment. Nevertheless, a subset will undergo further treatment with radiation.^15^ According to our results, the proportion of patients receiving postoperative RT has increased by over 80% from 2004 to 2016.

Previous studies have shown postoperative RT to be linked to improved disease outcomes after RP.^16^, ^17^ Specifically, three randomized trials (SWOG 8794, EORTC 22911, and ARO 96‐02) found reduction in biochemical recurrence of PSA with the use of postoperative RT in patients with high pathological or surgical risk features.^8^, ^9^, ^18^ Despite this, literature has shown a lack of utilization of adjuvant RT in these patients. In fact, the utilization of adjuvant RT with adverse features has been shown to be decreasing in recent years according to other studies.^10^, ^19^ We suspect slow uptake of postoperative RT is related to provider perception of poorer functional outcomes in patients who receive RT after RP.^20^ A post hoc analysis shows that decreased adjuvant RT utilization was no longer a trend in our extended study period from 2004‐2016, with 13.2% patients with adverse features receiving RT after RP in 2004, and 13.1% in 2016 (p_trend_ =0.370). Still, the consistent proportion of patients with adverse features receiving postoperative RT means that another population must account for the overall increase in postoperative RT among patients treated for PCa.

We conclude from our findings that the increase in RP as primary treatment for PCa is likely contributing to the increase in patients receiving postoperative RT. With the increase in RP, it appears clinically high‐risk patients may be increasingly selected for RP rather than RT. In our entire cohort of patients receiving curative treatment for their PCa, we found an overall increase in the utilization of RT after surgery. This increase in RT was seen in all treatment classifications, but the change in magnitude was greatest in the clinically high‐risk treatment group.

These changes in treatment patterns have been accompanied by an increase in active surveillance for low‐risk disease. As affirmed by findings from the ProtecT trial, active surveillance has become the preferred management strategy for patients with low‐risk PCa (see NCCN guidelines).^3^ In support of this trend, continued risk stratification of intermediate‐risk PCa patients also may be increasing the number of patients considered “favorable intermediate risk” being considered for active surveillance.^21^ It remains to be seen whether increased RP for high‐risk patients is simply coincidental or is indirectly related to increased rates of active surveillance among low‐risk patients.

Additionally, with the increase in postoperative RT demonstrated in our study, we hypothesize that although providers hope to treat patients with only RP, a likely sequela is a further delay in receiving postoperative RT. In surveys of urologists and radiation oncologists, urologists were shown to prefer delayed SRT, in comparison to radiation oncologists who were shown to prefer adjuvant RT or early salvage RT.^22^, ^23^ In support of this, immediate postoperative RT have been shown to be associated with worse functional outcomes for erectile dysfunction as well as urinary incontinence.^20^ Also, although randomized trials have shown more immediate RT after RP to best reduce the risks of local and biochemical recurrence in patients with adverse features, retrospective studies have suggested delayed salvage RT with observation can adequately control disease progression.^17^ Currently, there is not a randomized trial comparing early adjuvant or late salvage RT. Considering this discussion, urologists may weigh functional outcomes over oncologic outcomes in their decision to begin postoperative RT, increasing the time between surgery and receipt of radiation.

Of the variables associated with treatment, travel distance was the only one of the demographic and socioeconomic variables that had similar predictive odds ratios for selection of RT as primary treatment and selection of postoperative RT. This is unsurprising, considering RT regimens often involve multiple visits to the treatment location. The other variable that had a similar predictive value between postoperative RT and primary treatment with RT, was facility volume. Other studies have commented on the improved outcomes of RP for high‐volume treatment centers, and it is likely that improved surgical technique makes RP a more attractive option for primary therapy, as well as reducing the likelihood of high‐risk features that later indicate adjuvant RT.^24^

The increasing utilization of robotic surgery could also be a driver of increasing utilization of RP as a primary treatment choice. With robotic surgery, treatment centers have been able to significantly increase their capacity to treat patients surgically. One population‐based study showed surgeons who adopted robotic RP were able to increase their annual volume significantly, as high as an additional 14 RP cases a year in some groups.^25^ Although new RT technologies, such as proton beamy therapy, have also emerged, studies have shown that utilization of these therapies is more limited in comparison to robotic RP.^26^, ^27^

Although, the percentage of patients receiving postoperative RT in our study may be modest, when considering the number of patients receiving treatment year to year, the high incidence of PCa means that thousands of patients receive postoperative RT for their PCa treatment.^1^ Although some observational studies suggest there may be marginal disease benefits to choosing RP over RT as primary treatment, there is not a clinical trial showing therapeutic advantage to one therapy or the other.^28^, ^29^, ^30^ More careful patient selection of patients for primary treatment may help us increase the number of patients who undergo single‐modality therapy.

Limitations of our analysis include its retrospective, nonrandomized nature with potential confounding variables unable to be controlled for. Although we identified clinical factors to be most strongly associated with the receipt of postoperative treatment, our data cannot discount other causes such as the preferences of patients and providers. Possibly, patients may be increasingly preferring one‐day treatment with surgery rather than many sessions of RT. Considering that RT and RP are presumed to be similarly effective treatments, this would not be unreasonable, and is supported by the fact that patients with greater travel distance were more likely to get surgery. Although we hypothesize this increase in postoperative RT may be driven by the selection of clinically high‐risk patients for surgery, another limitation is the difficulty in knowing the exact reasons why patients were selected for radiation after surgery. Nevertheless, we are the first to comment on the increasing trend in the overall utilization of postoperative RT, and this seems to be driven by preoperative clinically high‐risk patients.

## 5 CONCLUSIONS

We saw an overall increase in the utilization of RT in patients who had already received RP between 2004 and 2016. This increase was primarily driven by patients who were clinically high risk before surgery, although increases were seen in all treatment groups. Our study suggests that careful selection of the primary treatment is needed for PCa patients, especially among clinically high‐risk patients. With improved selection, we may be able to increase the percentage of patients who receive single‐modality therapy for PCa.

## CONFLICT OF INTEREST

The authors have no financial or non‐financial competing interests to declare.

## AUTHORS’ CONTRIBUTIONS

All authors were involved in developing the principal research questions for this manuscript. Statistical data analysis was performed by BK. After statistical analysis, the initial draft of the manuscript was written by BK. After drafting, all authors read, edited, and then approved of the final manuscript.

## ETHICS APPROVAL AND CONSENT TO PARTICIPATE

Approval for the study was waived by the Yale Research Protection Program institutional review board.

## Data Availability

The data that support the findings of this study are available from the American College of Surgeons but restrictions apply to the availability of these data, which were used under license for this study, and so are not publicly available. Data are, however, available from the authors upon reasonable request and with permission of the American College of Surgeons.
